# Mr. Woodham in Reply to Mr. Walker

**Published:** 1817-01

**Authors:** 


*For the London Medical and Physical Journal.*

### Mr. Woodham *in Reply to Mr. Walker.*

"AN irresistible propensity to the *cacoethes scribendi*" forms the *fourth* charge (I have already answered three) which Mr. Walker has brought against me. To this I have to reply, that, admitting the habit of occasionally writing, particularly against *innate essences*, common-place observations and variolous journals, the accusation comes with a peculiarly peculiarly ill grace from one whose sesquipedalian essays so *regularly* present themselves to the readers of the Medical and Physical Journal.

Permit me, Mr. Editor, to avail myself of this opportunity to remark, that, though your Nestorean Correspondent's practical observations on small-pox are not accompanied by Sydenham's hypothesis, they are adorned by the equally-luminous one of his own; and, though disposed to pay every deference to your opinions, I cannot conceive that *any* practitioner, howsoever young, could be at a loss for the treatment of that most loathsome disease, when it were to be found in every system, compendium, and *vade-mecum*, of medical practice.

*London; Dec. 6.*

